# The Role of Gut Microbiota in the Onset and Progression of Obesity and Associated Comorbidities

**DOI:** 10.3390/ijms252212321

**Published:** 2024-11-16

**Authors:** Robert-Mihai Enache, Monica Profir, Oana Alexandra Roşu, Sanda Maria Creţoiu, Bogdan Severus Gaspar

**Affiliations:** 1 Department of Radiology and Medical Imaging, Fundeni Clinical Institute, 022328 Bucharest, Romania; robert-mihai.enache@rez.umfcd.ro; 2 Department of Morphological Sciences, Cell and Molecular Biology and Histology, Carol Davila University of Medicine and Pharmacy, 050474 Bucharest, Romania; monica.profir@rez.umfcd.ro (M.P.); oana-alexandra.rosu@rez.umfcd.ro (O.A.R.); 3 Department of Oncology, Elias University Emergency Hospital, 011461 Bucharest, Romania; 4 Department of Surgery, Carol Davila University of Medicine and Pharmacy, 050474 Bucharest, Romania; bogdan.gaspar@umfcd.ro; 5 Surgery Clinic, Bucharest Emergency Clinical Hospital, 014461 Bucharest, Romania

**Keywords:** obesity, microbiota, dysbiosis, gastrointestinal microbiome, diet, bariatric surgery, probiotics, prebiotics, postbiotics

## Abstract

Obesity, a global public health problem, is constantly increasing, so the concerns in preventing and combating it are increasingly focused on the intestinal microbiota. It was found that the microbiota is different in lean people compared to obese individuals, but the exact mechanisms by which energy homeostasis is influenced are still incompletely known. Numerous studies show the involvement of certain bacterial species in promoting obesity and associated diseases such as diabetes, hypertension, cancer, etc. Our aim is to summarize the main findings regarding the influence of several factors such as lifestyle changes, including diet and bariatric surgery, on the diversity of the gut microbiota in obese individuals. The second purpose of this paper is to investigate the potential effect of various microbiota modulation techniques on ameliorating obesity and its comorbidities. A literature search was conducted using the PubMed database, identifying articles published between 2019 and 2024. Most studies identified suggest that obesity is generally associated with alterations of the gut microbiome such as decreased microbial diversity, an increased Firmicutes-to-Bacteroidetes ratio, and increased SCFAs levels. Our findings also indicate that gut microbiota modulation techniques could represent a novel strategy in treating obesity and related metabolic diseases. Although some mechanisms (e.g., inflammation or hormonal regulation) are already considered a powerful connection between gut microbiota and obesity development, further research is needed to enhance the knowledge on this particular topic.

## 1. Introduction

Obesity represents a complex metabolic disorder influenced by both genetic and environmental factors. The World Health Organization (WHO) defines obesity as having a body mass index (BMI) over 30, but the definition can vary across countries. For example, WHO experts argue that the relationships between BMI, body fat percentage, and health risks vary between Asian and European populations. Thus, the WHO panel suggested a lower BMI threshold for defining obesity in Asian populations at ≥27.5 kg/m^2^, compared to the standard cutoff of ≥30.0 kg/m^2^ [1].

Approximately one-third of the world population is overweight, and around 10% are considered obese [2]. Due to several lifestyle, environmental, and genetic factors, obesity rates have been steadily rising for several years and are expected to increase more in the following years. Unfortunately, obesity has been determined to be a contributing key factor to the elevated rates of morbidity and mortality in the modern era [3].

Patients with obesity present an elevated risk of developing various illnesses, including type 2 diabetes (T2D), nonalcoholic fatty liver disease (NAFLD), other pathological conditions related to the liver, cardiovascular diseases, and certain cancers [4,5,6]. Thus, several researchers have focused on better understanding the mechanisms behind obesity and the comorbidities it is associated with. Increased attention has been given to the involvement of the gut microbiota in obesity and related metabolic diseases. The gut microbiome represents an ecosystem of commensal, symbiotic, and pathogenic microorganisms that reside within the intestinal lumen that play a key role in maintaining the general health of the host [7]. The gut microbiota is genetically influenced and continuously shaped from gestation and birth, throughout early childhood and into adulthood, being dependent on diet, physical activity, geographic location, exposure to xenobiotics, and other environmental factors [5,8]. During gestation and birth, gut microbiota colonization occurs primarily through mother-to-infant transmission, with the newborn’s gut microbiota predominantly composed of *Enterococcus*, *Escherichia/Shigella*, *Streptococcus*, and *Rothia*, while *Bifidobacterium* and *Collinsella* are more common in infants [4,9,10,11]. However, recent studies utilizing metagenomic methods highlighted that the gut microbiota of newborns is very different than that of infants, with full microbiota establishment not occurring until at least two years of age, thus describing many differences in microbiota composition between 4-month-old infants (where the dominating bacteria are *Bifidobacterium*, *Lactobacillus*, *Collinsella*, *Granulicatella*, and *Veillonella*) and 12-month-old infants, whose microbiota more closely resembles that of adults [12,13].

The diet provided to a child is crucial for modulating and establishing a mature gut microbiota, which has a major impact on how genes are expressed in relation to vitamin biosynthesis and polysaccharide digestion [14]. The gut microbiota can trigger obesity and other disorders by influencing host gene expression and inducing a variety of epigenetic modifications, including DNA methylation, histone and chromatin modifications, and regulation of non-coding RNA (ncRNA) [15]. These epigenetic changes are mediated by metabolites generated by the gut microbiota, such as trimethylamine N-oxide, folates, biotin, and short-chain fatty acids [16]. Notably, research suggests that these epigenetic mechanisms can help uncover the underlying causes of various diseases [9].

Gut microbiota has become a focal point in recent research due to its significant contribution in obesity and associated comorbidities. It influences adiposity and glucose metabolism, leading to obesity through dysbiosis. Additionally, it offers a potential therapeutic avenue for managing obesity, particularly through dietary interventions [5,17].

A high-caloric diet, particularly one high in lipids, combined with sedentary behavior, is the primary risk factor for obesity and its related pathological conditions. These variables cause the body to store extra energy as fat and cause lipid buildup in visceral and subcutaneous adipose tissue. When adipose tissue can no longer store surplus energy as triglycerides, lipids increase in the bloodstream and accumulate in non-adipose tissues, leading to ectopic fat storage [18]. Pro-inflammatory cytokines and adipokines are produced and released as a result of this process, and they play a major role in the development of disorders linked to obesity [5,19].

The gut microbiota has become a focal point in recent research due to its significant role in obesity and associated comorbidities by being related to many processes in the human body, its influence spreading much further than the gastrointestinal system [20]. It is thought that the imbalance of the gut microbiota species and their diversity—known as dysbiosis—is responsible for the activation of many signals through different pathways, leading to obesity and related complications.

Dysbiosis plays an important role in increasing adiposity, inflammatory responses, oxidative stress, and metabolic dysfunction. Dysbiosis also affects glucose metabolism and the distributions of adipocytic cells in the body. Furthermore, compared to people of average height and weight, the gut microbiota of obese people is more successful at obtaining energy from food [5]. Therefore, medical and non-medical interventions to halt the progression of obesity are crucial for controlling the onset of related diseases and improving public health. Unfortunately, the development of effective screening, diagnostic, and management strategies for obesity and its comorbidities remains limited due to gaps in understanding its underlying pathophysiology [5].

Research has demonstrated that a diet very high in fat can reduce the levels of Gram-negative bacteria, such as *Bacteroides*-related bacteria, and Gram-positive bacteria, like *Eubacterium rectale*, *Clostridium coccoides*, and *Bifidobacterium*, in obese people. However, these changes can be reversed through dietary interventions [21,22]. Thingholm et al. observed that *Lactobacillus reuteri* is more abundant in the gut microbiota of obese patients, while *Akkermansia*, *Faecalibacterium*, *Oscillibacter*, *Alistipes*, and *Methanobrevibacter smithii* tend to be insufficient, highlighting the value of these microbiota shifts as early diagnostic markers for managing T2D [23].

For instance, the *Prevotella* population increases in the gut microbiota of patients who are consuming more fibers, while *Bacteroides* flourish in diets that are based on proteins, both of which help reduce chronic systemic inflammation. Some nutrients have been associated with beneficial impacts on the gut microbiota through the restoration of the *Firmicutes*/*Bacteroidetes* ratio, including but not limited to coffee, green tea, omega-3 polyunsaturated fatty acids, and fruits and vegetables [24,25].

Probiotics including *Bifidobacterium* and *Lactobacillus* and prebiotics (such as lactulose, inulin, fructooligosaccharides, and galactooligosaccharides) found in the Mediterranean diet are also effective strategies for treating dysbiosis [5]. Conversely, adults consuming a Western diet show a heightened risk of obesity and related conditions like coronary vascular disease, metabolic syndrome, and NAFLD [26].

Fecal microbiota transplantation (FMT) is an additional promising strategy currently used in many obese patients, improving gut microbiota diversity and enhancing insulin sensitivity [27]. Moreover, studies suggest a potential role for antibiotics, particularly vancomycin, in treating dysbiosis related to obesity by reducing TNF-α levels in mice and increasing insulin sensitivity in humans [28,29].

Another promising strategy for treating obesity and related diseases is brown adipocyte thermogenesis. This approach stimulates the production of acetate and lactate, activating adipose tissue browning, thereby promoting fat burning [30]. However, the key challenge for future development is identifying a pharmacological method to reliably induce thermogenesis in the human body, which could significantly enhance obesity treatment outcomes [31].

Bariatric surgery is recognized as an important and effective tool for obesity and its comorbidities, especially when other therapies have been unsuccessful. It has long-term impacts on the gut microbiota in addition to its immediate advantages, which could help patients to maintain a healthy weight [5,32,33,34].

The benefits of bariatric surgery on gut microbiota may stem from several factors, including reduced gastric volume, increased luminal pH, and less efficient energy extraction from the diet. These changes typically result in a decrease in *Firmicutes* (high concentrations are associated with obesity), an increase in *Proteobacteria* (which positively affects systemic inflammation, glucose homeostasis, and weight loss), and an increase in *Enterobacteriaceae* (which is negatively correlated with cholesterol levels and positively correlated with weight loss). Additionally, there is a decrease in *Clostridiaceae* and *Lachnospiraceae*, which are related to weight loss [34].

However, weight regain remains a significant challenge, with studies indicating that approximately one-third of patients experience excessive weight regain after the surgery [34,35].

**Figure 1:**
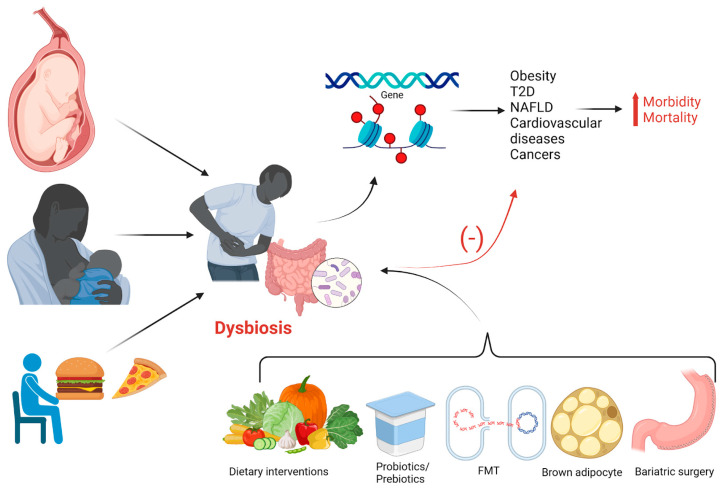
The gut microbiota is shaped from gestation and birth (via mother-to-infant transmission), through early childhood (influenced by diet), and into adulthood (affected by diet and lifestyle). This process could lead to dysbiosis, particularly with high-calorie, fat-rich diets and sedentary behavior. In the case of dysbiosis, the gut microbiota can influence host gene expression and trigger various epigenetic changes, contributing to obesity and related comorbidities and increasing morbidity and mortality. However, dietary and non-dietary interventions can help restore a balanced microbiota, reducing the risk of obesity-related comorbidities. Created with BioRender.com (accessed on 7 September 2024).

A summary of this chapter is presented in Figure 1.

The main findings of this review are the alterations of the gut microbial composition such as decreased microbial diversity, an increased F/B ratio, and alterations in the abundance of several specific bacteria associated with obesity. These findings are consistent with the conclusions of previous reviews [36]. Similar to previous studies, we aim to underline the relationship between the gut microbiome and obesity-related inflammation and how obesity is linked to diet, the gut microbiota, and its metabolites [37]. This review summarizes the main findings and attempts to identify methods to restore the balance of the disturbed microbiota through lifestyle changes, including diet, bariatric surgery to reduce weight, and the administration of prebiotics, probiotics, and postbiotics. The gut microbiota is thought to provide a novel viewpoint on possible therapies by establishing a connection between inflammation, energy homeostasis, metabolism, and obesity. The role of microbiota modulation techniques has been previously discussed by other authors [36]. Our aim is to identify novel studies that bring more clarity to this subject. On the other hand, the current review discusses comorbidities related to obesity, highlighting trends in this area and advancing our knowledge on obesity and its long-term effects on the homeostasis of human health. Given how quickly this field of study is developing, the growing interest in gut microbiota-targeted therapeutics covered in the current paper has the potential to both broaden and pave the way for the identification of interesting areas for further investigation and development. The current analysis included information concerning diet, environmental factors, and physical activity because they were thought to be relevant for a more integrated approach to obesity and its comorbidities. In an effort to improve understanding and advance education, the purpose of this review is to help discover gaps in the currently known research. The gut microbiota offers a potential therapeutic avenue for managing obesity since it is very susceptible to lifestyle changes, and a thorough knowledge can guide future studies for a fresh perspective on the subject.

## 2. Methods

To achieve the desired results, an initial search of specialized scientific publications was conducted using PubMed. Articles were selected based on contemporary data, both in vivo and in vitro, with the majority focusing on human subjects but some on rodents, utilizing the keywords: “obesity”, „gut microbiota”, „dysbiosis”, „bariatric surgery”, and „probiotics”. For an advanced selection of bibliographic sources, the following filters were applied: full-text articles, articles in English, published from 2019 to 2024, and excluding preprints. Review articles were also included in the search and cited in our work. After a preliminary analysis of topic relevance, original articles, randomized clinical trials, meta-analyses, and review articles containing up-to-date information and contemporary concepts on gut microbiota alteration in obese patients and the mechanisms involved in this process were selected. The focus was specifically on obese adults, their dysbiosis, and their risk of developing obesity-related comorbidities. Nevertheless, for a broader view of the issue, we also searched for information about specific subgroups: such as pregnant women, or children under 10 years old. We included some of these studies in Table 1 in our attempt to provide a comprehensive overview of the scientific literature from the past five years. This table summarizes the most relevant methodological approaches employed and the conclusions and limitations drawn from the reviewed research.

## 3. Genetics, Epigenetics, and Gut Microbiota in Obesity

A person’s diet and genetic and epigenetic background are major determinants of changes in the composition of their gut microbiota, which can impact the development of obesity and associated comorbidities [8,46]. In dysbiosis, the altered microbiota produces microbially derived metabolites like short-chain fatty acids (SCFAs), which can be considered an additional energy source. Dysbiosis also leads to an overgrowth of Gram-negative bacteria, increasing gut permeability and facilitating bacterial lipopolysaccharide (LPS) translocation, which triggers endotoxemia and low-grade systemic inflammation. These changes can result in genetic and epigenetic alterations that elevate the risk of obesity and its related comorbidities [47].

Research has shown that different bacterial species in the human gut microbiota possess distinct gene types and can both influence metabolic phenotypes [47,48]. A metagenomic study by Li et al. analyzed the gut microbiota of 192 patients and found that Bacteroides and *Prevotella* displayed functional gene differences related to amino acid and carbohydrate metabolism. They also identified a human genetic variant (rs878394) linked to the gene lysophospholipase-like 1 (LYPLAL1), which is associated with body fat distribution and insulin sensitivity, and the abundance of *Prevotella*. Moreover, the study found that patients with a predominance of *Bacteroides* had diets rich in protein and animal fat, while those with a predominance of *Prevotella* had carbohydrate-rich diets [49].

Another study by Richards et al. investigated the transcriptional changes in human colonic epithelial cells caused by fecal microbiota in an experimental in vivo study. The researchers identified over 6000 genes, including a 1.8-fold enrichment in genes and single-nucleotide polymorphisms (SNPs) across various genes (e.g., USP36, PIP5K1A, AFAP1L2, GIPC1, ASAP2, RNF213, KCTD12, and LASP1), which were associated with microbiome-related diseases such as obesity, colorectal cancer, and T2D (Fisher’s exact test *p* = 0.03; OR = 1.5) [50].

In a separate experimental study, Davenport et al. examined the association between 200,000 host genotypes and the relative abundance of fecal bacterial taxa. They identified at least eight bacterial taxa whose abundances were linked to SNPs, including *Akkermansia*, which was associated with the gene phospholipase D1 (PLD1), a gene linked to body mass index [51].

The colonization and development of a child’s gut microbiota has been demonstrated to be significantly influenced by epigenetic mechanisms, which are intimately related to variables like birth mode, nursing, diet variety, illnesses, and antibiotic use [47]. For instance, by blocking histone deacetylase activity and controlling histone acetylation and methylation, SCFAs can alter the expression of genes related to cellular lipid metabolism and restore chromatin modification states. Furthermore, Western-style diets may shield against microbiota-induced chromatin modifications, which are usually noticed following a diet high in polysaccharides [8,47].

DNA methylation is an epigenetic mechanism that regulates the accessibility of transcription factors, histone modifiers, and the transcriptional machinery to chromatin. This process is made possible by enzymes known as DNA methyltransferases (DNMTs) [52]. SCFAs, metabolites produced in the gut, can impact DNA methylation by activating the MAP kinase pathway and reducing DNMT1 activity, leading to the demethylation of tumor suppressor genes such as RARB2, p16, and p21 [53]. Butyrate-producing bacteria, like *Clostridium*, play a crucial role in generating gut metabolites that help regulate diabetes and obesity [8]. Additionally, other studies have suggested a correlation between DNA methylation and tumorigenesis in colorectal cancer patients with a specific mutational profile, as well as an association with *Fusobacterium nucleatum* [52].

Studies have revealed that the microbiota of obese people is characterized by decreased methylation of the FFAR3 gene, decreased microbial diversity, and a lower quantity of *Faecalibacterium prausnitzii*. Additionally, the ratio of *Firmicutes* to *Bacteroidetes* has shifted, and the TLR4 and TLR2 genes, all of which are connected with body mass index (BMI), have less methylation [47]. In a pilot study, Kumar et al. evaluated the DNA methylomes of eight pregnant women (BMI ≤ 25), putting them into groups according to the main microbiota they had (*Proteobacteria* and *Bacteroidetes*/*Firmicutes*). The findings demonstrated a relationship between epigenetic patterns and bacterial predominance [54].

ncRNAs are RNA transcripts that do not code for or translate into proteins [55]. The major types of ncRNAs include long non-coding RNAs (lncRNAs), short interfering RNAs (siRNAs), and microRNAs (miRNAs). Alterations in ncRNAs can contribute to various diseases, including obesity and related conditions. miRNAs, the most common type of ncRNA, can affect the composition of the gut microbiome and are crucial in maintaining metabolic homeostasis through a bidirectional interaction between the host and microbiome. Their dysregulation may elevate the risk of insulin resistance and obesity [8,35].

An in vivo study by Virtue et al. analyzed miRNA expression in miR–181-deficient mice on a normal chow diet compared to obese mice on a high-fat diet. The study found that miR-181 was upregulated in epididymal white adipose tissue during diet-induced obesity, and miR–181-deficient mice were fully protected from developing obesity, indicating that miR-181 plays a role in obesity and related comorbidities. The researchers also assessed blood insulin levels, glucose, and insulin tolerance in lean and obese mice, observing that lean miR–181-deficient mice were more insulin-sensitive and protected against obesity. In obese mice fed a high-fat diet, changes in gut microbiota composition were noted, particularly a decrease in the indole and its derivatives, which can dysregulate miR-181 in white adipose tissue adipocytes and contribute to obesity. The study highlighted that specific microbiota-derived metabolites regulate miR-181 expression in white adipocytes, impacting energy expenditure, adiposity, inflammation, and insulin sensitivity. Thus, targeting the microbiota–miRNA axis could be a potential mechanism for treating obesity [39].

Therefore, dietary interventions aimed at promoting beneficial bacterial populations and influencing epigenetic changes related to energy homeostasis could serve as potential preventive therapies for obesity and its related comorbidities [47].

**Figure 2:**
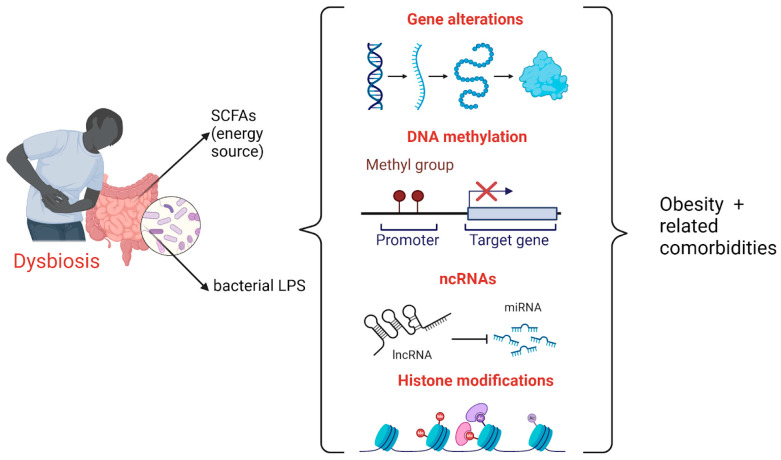
In cases of dysbiosis, the levels of metabolites such as SCFAs and translocated bacterial lipopolysaccharides increase, leading to genetic alterations and epigenetic modifications (including DNA methylation, ncRNAs, and histone changes), which heighten the risk of obesity and its related comorbidities. Created with BioRender.com (accessed on 8 September 2024).

Figure 2 provides a summary of the link between gene expression, epigenetic modifications, and microbiome in the development of obesity and its related comorbidities.

## 4. Importance of Gut Microbiota and Diet in Obesity

An animal in vivo study by Bäckhed et al. was among the first to demonstrate the critical role the gut microbiota plays in obesity [56]. The authors demonstrated that, despite the decreased food intake, the transplantation of a normal intestinal microbiota from conventionally grown rodents into germ-free mice resulted in an increase in body fat and insulin resistance. This specific study also showed that intestinal lipoprotein lipase inhibitors are suppressed by the gut microbiota, which encourages triglyceride storage in adipocytes [56].

The consumption of a high-fat, high-sugar, low-fiber diet plays the main role in obesity and metabolic disorders [57]. The question of whether diet affects weight increase or not is rarely disputed; however, it is still unclear how diet affects weight loss by altering the gut flora. Research has indicated that changes in gut microbiota and elevated intestinal inflammation occur prior to weight gain and its associated metabolic effects [58]. The intestinal microbiota composition is highly dependent on dietary habits and the observed changes might be because of the capacity of different bacterial species to use certain substances [59].

For example, a high-fat diet leads to increased *Firmicutes* abundance and decreased *Bacteroidetes* abundance in the gut, increasing the *Firmicutes*-to-*Bacteroidetes* (F/B) ratio [60]. An altered F/B ratio has been observed in obese animal models with a leptin gene mutation as opposed to their lean siblings who did not express the mutations, suggesting that obesity influences gut microbial diversity [61]. Increased energy source metabolic breakdown has been linked to elevated *Firmicutes* levels, which increase calorie intake and cause weight gain [59]. High-fat diets also cause an increase in bacteria linked to the secretion of pro-inflammatory chemicals, which compromise the integrity of the gut barrier, and a decrease in the *Lactobacillus* genus abundance [58].

An increased intake of sugar leads to dysbiosis by increasing the relative abundance of intestinal *Proteobacteria* and decreasing the abundance of *Bacteroidetes*, which has pro-inflammatory effects and lowers epithelial barrier function [62]. Animal studies have shown that these changes, increased inflammation and intestinal permeability, lead to endotoxemia, increased fat storage, and steatosis [63].

Consuming fiber is also essential for preserving host health and managing weight. The gut microbiota generates metabolites like butyrate and propionate through the microbial metabolism of ingested fiber [64]. SCFAs (butyrate, propionate, and acetate) regulate glucose and lipid metabolism and the sensation of satiety via activation of certain proteins, thus being involved in the pathogenesis of obesity [65]. Butyrate and propionate control the sensation of satiety by increasing the production and secretion of GLP-1, which interacts with the pancreatic β-cells, leading to increased insulin secretion and decreased glucagon secretion [66]. In addition, butyrate and propionate can also increase histone acetylation via inhibition of histone deacetylase activity and expression [67]. SCFAs mediate lipid metabolism through G-protein-coupled receptors (GPRs), mainly GRP41 and GPR43 and via activation of the peroxisome proliferator-activated receptor (PPAR-γ) [17,68]. An in vivo study has shown that propionic acid directly affects adipose tissue through the inhibition of inflammatory cytokines and chemokines, as well as the promotion of lipogenesis and improved uptake of glucose [69].

Most studies agree on the fact that the microbiota of obese patients has a decreased microbial diversity, which is associated with insulin resistance and inflammation [59]. Several studies have sought to characterize the gut microbiota of obese adults and to find what differentiates it from the microbiota of healthy, normal-weight people. One of the main findings is a decrease in the abundance of *Akkermansia muciniphila* in obese and overweight patients [40]. *A. muncipila* is a mucin-degrading bacterium belonging to the Verrucomicrobia phylum, which plays an essential role in weight loss. It has been observed to reduce insulin resistance and decrease steatosis and excess weight in rodents [70].

Although several studies have reported differences in the F/B ratio in obese adults, other authors suggest that obesity is associated with an increased abundance of the *Actinobacteria* and *Firmicutes* phyla and a decreased abundance of species of *Bacteroidetes*, *Verrucomicrobia*, and *Faecalibacterium prausnitzii* [71].

An in vivo study conducted on obese adolescents who underwent a diet low in calories and increased physical activity showed that dietary treatments aimed at weight loss caused alterations in the makeup of gut microbiota. The authors have reported increased levels of the genus *Bacteroides*, *Lactobacillus*, and *Prevotella*, along with a decrease in the levels of the genus *Bifidobacterium*, *Clostridium*, *Eubacterium*, and *Akkermansia* [72]. Moreover, the observation made by Ley et al. that a low-calorie diet leads to an increased proportion of *Bacteroidetes* suggests that interventions to the gut microbiota can positively influence weight loss [22].

## 5. Dysbiosis and Correlations with Obesity

The gut microbiota produces numerous microbial metabolites, components, peptides, and proteins and are involved in several physiological and pathological processes, such as inflammation, insulin resistance, homeostasis, and obesity. Dysbiosis refers to disrupting the normal microbiota, resulting in an abundance of dangerous bacteria and contributing to various diseases associated with gut health [68,73]. Thus, dysbiosis, meaning an imbalance in the gut microbiota, may be a factor in obesity, fat accumulation, and associated comorbidities [17,74]. Modern technologies are now available everywhere, including metabolomics, metagenomics, meta-transcriptomics, and meta-proteomics. By analyzing bacterial genes obtained from intestinal biopsies or fecal samples, these approaches highlight the critical relationship between host physiology and the gut microbiota [74,75].

In an experimental in vitro study by Ley et al. the researchers examined 5088 bacterial 16S rRNA gene sequences from the distal gut microbiota of genetically obese, lean, and wild-type mice, as well as their obese mothers, all fed a diet rich in polysaccharides. The study revealed that gut microbiota composition is maternally transmitted, with obese mice showing a 50% reduction in *Bacteroidetes* and a significantly higher proportion of *Firmicutes* (*p* < 0.05). Additionally, the obese mice consumed more food compared to lean mice (*p* < 0.001), leading to a higher BMI (34.5 ± 3.4 g vs. 19.1 ± 1.9 g at 8 weeks; *p* < 0.001) [61].

In a comparative study, Turnbaugh et al. used shotgun metagenomics sequencing to examine obese mice and humans. They observed a larger *Firmicutes*-to-*Bacteroidetes* ratio, a substantial rise in *Archaea* in obese mice (*p* < 0.001), and an increased ability to extract energy from the diet. Additionally, other studies have linked obesity with elevated levels of *Halomonas* and *Sphingomonas* bacteria, and a reduction in *Bifidobacteria* [76,77]

In a literature review by Stojanov et al., the authors emphasized that a higher *Firmicutes*-to-*Bacteroidetes* ratio is linked to obesity [78]. Further studies have shown that the gut microbiota of germ-free mice can be influenced by the gut microbiota of either obese or lean mice, resulting in an increase in total body fat of 47% and 27%, respectively [76]. An in vivo study by Kasai et al. examined 23 non-obese (BMI < 20 kg/m^2^) and 33 obese (BMI ≥ 25 kg/m^2^) subjects. According to their findings, those who were fat had higher levels of bacterial diversity (*p* < 0.05), a drop in *Bacteroidetes* (23.28% in obese vs. 35.44% in non-obese; *p* < 0.05), and an increase in the ratio of Firmicutes to *Bacteroidetes*. Certain bacterial species, including *Blautia hydrogenotrophica*, *Coprococcus catus*, *Eubacterium ventriosum*, *Ruminococcus bromii*, and *Ruminococcus obeum*, were significantly associated with obesity [79]. Similarly, in a review by Cani et al., the authors noted that members of the *Firmicutes* family, such as *Clostridium*, *Lactobacillus*, and *Ruminococcus*, are elevated in obese patients, while *Faecalibacterium prausnitzii* is more prevalent in healthy patients and reduced in those with obesity [80]. Koliada et al. conducted a metagenomic analysis of 61 Ukrainian adults, showing that *Firmicutes* levels increased with BMI, while *Bacteroidetes* levels decreased. The rise in the *Firmicutes*-to-*Bacteroidetes* ratio was strongly linked to BMI (OR = 1.23, 95% CI 1.09–1.38), with patients having a ratio of ≥1 being 23% more likely to be overweight [81]. Collectively, these studies suggest a significant role of gut bacteria in the development of obesity [74]. The processes by which gut microbiota and dysbiosis contribute to the pathophysiology of obesity are extensively documented in several research. These mechanisms include the synthesis of SCFAs, connections to low-grade inflammation, control over fat deposition, and bile acid metabolism [74].

SCFAs, including acetate, butyrate, lactate, and propionate, are metabolites produced in the colon by gut microbiota, particularly fibrolytic bacteria such as *Bacteroides*, *Roseburia*, *Ruminococcus*, *Bifidobacterium*, *Lactobacillus*, and *Eubacterium*, through fermentation using hydrolytic enzymes [75,82,83]. As previously mentioned, the gut microbiota in obese patients have an enhanced ability to extract energy from the diet, generating additional calories and SCFAs through catabolic genes, potentially contributing to weight gain [84,85]. Elevated SCFA levels can lead to dysbiosis by lowering luminal pH, promoting the growth of butyrate-producing bacteria that create anaerobic conditions by supplying energy to colonocytes and consuming oxygen. These changes can also modulate the pro-inflammatory activation of epithelial and myeloid cells [74,86].

Under normal conditions, SCFAs act as ligands for G-protein-coupled receptors (GPRs), with acetate binding to GPR43, butyrate binding to GPR41, and propionate binding to both. In cases of dysbiosis and obesity, these receptors may be downregulated, leading to hepatic lipogenesis and energy imbalance [87,88,89]. Studies have shown that SCFA levels are elevated in obese patients, with increased acetate in blood and feces (SMD = 0.87, 95% CI = 0.24–1.50), butyrate in feces (SMD = 0.78, 95% CI = 0.29–1.27), and propionate in feces (SMD = 0.86, 95% CI = 0.35–1.36) [74].

Acetate and propionate have been shown to increase fat oxidation and energy expenditure while decreasing lipolysis, thereby influencing lipid metabolism and glucose homeostasis, which also leads to improved insulin sensitivity, a function shared by butyrate [90]. Lower levels of anorexigenic hormones that promote satiety, such as glucagon-like peptide-1 (GLP-1) and peptide YY (PYY), have been observed in obese patients, which is another area influenced by SCFAs [74]. Acetate also affects the hypothalamus by upregulating the mRNA expression of α-melanocyte-stimulating hormone and downregulating the mRNA expression of agouti-related peptide. This regulation has been linked to the increased presence of certain bacteria in obese patients, such as *Bacteroides* and *Prevotella* [91,92].

Low-grade inflammation, which contributes to the development of obesity and its related disorders, such as insulin resistance, cardiovascular disease, and T2D, can be brought on by gut dysbiosis [74,93,94]. Gut dysbiosis-related inflammation is mediated by multiple mechanisms, one of which is the release of lipopolysaccharides (LPSs) by Gram-negative bacteria. LPS can enter chylomicrons or pass through damaged tight junctions to penetrate the intestinal barrier. Furthermore, disruption of tight junction proteins by a high-fat diet can lead to increased permeability of LPS [95]. After entering the bloodstream, LPS attaches to LPS-binding protein (LBP), stimulating the CD14 receptor that then engages in interaction with adipose tissue macrophages via toll-like receptor 4 (TLR4). Increased expression of genes encoding pro-inflammatory factors, such as nuclear factor kappa B (NF-κB), is the result of this activation [96].

Peroxisome proliferator-activated receptors (PPARs) are essential for controlling adipogenesis, and SCFAs have the ability to increase their expression [97]. An in vivo study by Rumberger et al. demonstrated that butyrate and propionate inhibit histone deacetylase, thereby increasing the rate of lipolysis in vitro [98]. Another in vitro study by Bäckhed et al. found that germ-free mice fed a high-fat diet had elevated levels of phosphorylated adenosine monophosphate-activated kinase (AMPK), a crucial enzyme in energy homeostasis and fatty acid oxidation, in the liver and skeletal muscles. These mice gained significantly less weight compared to conventionalized mice [99]. On the other hand, decreased phosphorylated AMPK levels might cause an increase in the synthesis of triglycerides and cholesterol, which can promote lipogenesis and obesity [100]. By blocking lipoprotein lipase and hence restricting triglyceride buildup in adipose tissue, the fasting-induced adipose factor (FIAF), a protein produced by adipose tissue, liver, skeletal muscle, and the gut in response to fasting, also plays a role in how fat is metabolized [56].

Hepatocytes produce bile acids, which are conjugated with taurine or glycine and secreted in bile. Examples of these bile acids are cholic acid and chenodeoxycholic acid [74]. Cholic acid and chenodeoxycholic acid are converted to form secondary bile acids, such as deoxycholic acid and lithocholic acid. The nuclear farnesoid X receptor (FXR), which is involved in the metabolism of glucose and lipids in the liver, can bind to these bile acids. Fatigue, insulin resistance, and NAFLD are among the conditions that may result from FXR activation’s role in metabolic liver dysfunction [74,101]. Additionally, bile acids can activate the G-protein-coupled bile acid receptor 1 (TGR5), which stimulates the release of GLP-1, aiding in glucose homeostasis [102].

## 6. Bariatric Surgery and the Impact on Gut Microbiota

Surgical methods used to assist people with extreme obesity in losing weight are referred to as bariatric surgery. The three most popular kinds are adjustable gastric banding, sleeve gastrectomy, and gastric bypass. These surgical procedures limit the amount of food that can be consumed and shrink the stomach. In addition to its cosmetic benefits, bariatric surgery is recognized as the most successful and long-lasting treatment for type 2 diabetes [103,104]. Blood glucose levels were lower in patients who underwent bariatric surgery prior to achieving weight loss. Recent studies suggest that the mechanism leading to the resolution of T2D is dependent on GLP1, which initiates insulin secretion after bariatric surgery [105].

Bariatric surgery has been reported to have long-term effects on the gut microbiota composition. Numerous studies have demonstrated that bariatric surgery improves microbial diversity [106,107,108]. A randomized controlled trial by Tremaroli et al. has demonstrated that fecal transplantation from bariatric surgery patients into germ-free mice has led to less weight gain compared to fecal transplantation from obese patients, demonstrating the causal relationship between the gut microbiota composition and obesity [109].

One effect of bariatric surgery reported by several authors is a reduction in the abundance of the *Firmicutes* phylum and an increase in the abundance of *Proteobacteria* [41,110,111]. As previously mentioned, increased levels of the *Firmicutes* phylum are associated with obesity due to an increased degradation of energy sources, which leads to a higher caloric absorption and weight gain, whereas the *Proteobacteria* phylum has been shown to have a positive effect due to its capacity to lower inflammation and regulate glucose metabolism [112,113]. This outcome is due to an increase in pH following bariatric surgery in the intestinal lumen and increased oxygen levels, inhibiting the growth of anaerobic bacteria and promoting the development of aerobic bacteria such as *Proteobacteria* [114].

One long-term longitudinal study that analyzed the fecal microbiome of obese patients before and after bariatric surgery reported increased detection of the genera *Butyricimonas*, *Parabacteroides*, and *Slackia* after the intervention. In contrast, the genera *Acinetobacter*, *Coprococcus*, *Lachnospira*, *Lactococcus*, *Megamonas*, *Oribacterium*, and *Phascolarctobacterium*, which were prevalent in non-operated obese patients, significantly decreased following the surgical procedure [34].

Interestingly, patients who yielded better results, with greater weight loss and weight loss maintenance, presented a higher diversity of core microbiota. These patients have higher levels of the *Sarcina*, *Butyrivibrio*, *Alkaliphilus*, *Lachnospira*, *Pseudoalteromonas*, and *Cetobacterium* genera [38].

The fecal metabolome analysis of obese patients after bariatric surgery has shown that the surgical intervention also modifies the metabolome profile. Yu et al. observed a decrease in caffeine metabolite, stress markers, indoles, nucleotides, and butyrate levels, in their in vivo pilot study [41]. Juarez-Fernandez et al. reported increased levels of methyl acetoacetate, carbamoyl aspartate, and serine phosphate after bariatric surgery and decreased levels of 5-aminolevulinic acid, taurine, and trimethylamine N-oxide (TMAO) [34]. Taurine is a metabolite involved in bile acid formation and increased concentrations have been previously associated with obesity [115]. TMAOs have been correlated to cardiovascular disease. Thus, a decrease in the level of TMAOs is clearly beneficial to general health [116].

Increased levels of SCFAs have also been reported in obese patients, correlated with increased energy extraction and higher caloric intake [117]. Animal studies have demonstrated that the administration of SCFAs has a positive outcome in obese subjects, reducing the inflammation associated with obesity and ameliorating insulin resistance [118,119,120]. In humans, higher levels of microbiota-produced SCFAs have been associated with improved insulin response after an oral glucose tolerance test [121]. The analysis of the metabolome of obese patients who underwent bariatric surgery has demonstrated that the levels of butyrate, acetate, and propionate significantly decrease following the surgical intervention [34,122,123].

## 7. Obesity Comorbidities and Gut Microbiota Modulation

Obesity has a negative impact on general health and is associated with several comorbidities such as cardiovascular diseases, T2D, dyslipidemia, respiratory problems (e.g., sleep apnea), gastrointestinal disorders (e.g., gastroesophageal reflux disease, NAFLD), and endocrine and reproductive disorders (polycystic ovarian syndrome, infertility) [124,125]. In addition, obesity increases the risk of developing cancer in different organs such as the breast, colon, rectum, uterus (specifically in the endometrium), liver, gallbladder, and pancreas [126]. The consequences obesity has on the cardiovascular system include hypertension, coronary artery disease, venous thromboembolism, heart failure, and stroke. These illnesses are linked to higher rates of morbidity and death and arise from intricate pathways [127]. T2D is a common complication of diabetes, and T2D and obesity present similar trends in terms of prevalence and incidence [128]. The primary mechanism is insulin resistance, which releases esterified fatty acids (NEFAs) into the system, further leading to inflammation and endothelial dysfunction [129]. In addition, the majority of obese patients have comorbid dyslipidemia with elevated LDL-cholesterol and reduced HDL-cholesterol due to an increased intake of lipids and a reduced capacity to metabolize triglycerides [130].

Metabolic syndrome refers to the combination of physiological, metabolic, and biochemical factors that contribute to conditions like cardiovascular disease and T2D. An increased waist circumference over 94 cm in men and over 80 cm in women, along with two of the following, are necessary for the diagnosis of the metabolic syndrome: increased blood glucose (>100 mg/mL) or diagnosed T2D; decreased HDL cholesterol (<40 mg/dL in men and <50 mg/dL in women) or drug treatment for low HDL cholesterol; increased blood triglycerides (>150 mg/d) or drug treatment for elevated triglycerides; and high blood pressure (>130/85 mmHg) or drug treatment for hypertension [131]. The main cardiovascular risk factors linked to metabolic syndrome include hypertension, obesity, dyslipidemia, elevated triglycerides, and insulin resistance [123]. Nevertheless, about 30% of all obese adults are also metabolically healthy. These patients exhibit elevated insulin sensitivity without the presence of hypertension, hyperlipidemia, or other hallmarks of the metabolic syndrome [131,132].

Gut microbiota modulation is a valid therapeutic option to prevent or treat obesity and its related comorbidities.

One way of modulating the gut microbiome is by administering probiotics, defined as “live microorganisms which, when consumed in adequate amounts, confer a health effect on the host” [133]. Researchers have demonstrated that probiotics can stimulate weight loss in obese animals and the administration of the *Lactobacillus* species has been studied the most. *Lactobacillus gasseri* BNR17 demonstrated “anti-obesity” and “anti-diabetes” effects, by suppressing fat gain and reducing fasting glycemia in obese mice [134,135]. Yun et al. also demonstrated in vivo that *L. gasseri* BNR17 can suppress glucose levels in the blood and ameliorate symptoms of diabetes in db/db mice [134]. *L. gasseri* SBT2055 has also been shown to reduce mice’s fat mass and adipocyte size [136,137]. One systematic review identified nine studies that showed *Lactobacillus* strains’ beneficial effects on weight loss when combined with a hypocaloric diet [138].

In addition to reducing weight gain, *L. rhamnosus* GG and *L. sakei* NR28 can also reduce lipogenic gene expression in rodents [139].

The administration of *Akkermansia muciniphila* was also proven effective in reducing body weight and downregulating pro-inflammatory cytokines (TNF-α, IL-1β, and IL-6) as well as in lowering blood lipid levels and ameliorating hepatic steatosis [140,141].

The beneficial influence of probiotics in weight loss has also been demonstrated in clinical trials. For example, supplementation with *L*. *plantarum* KY1032 and *L*. *curvatus* was shown to reduce body weight in overweight patients [43,142]. It has recently been demonstrated that a multistrain probiotic supplement combining strains of *Lactobacillus* and *Bifidobacterium* can cause weight loss, particularly the lowering of visceral fat, and can also decrease systemic inflammation [45]. In addition, the capacity of probiotics to regulate HDL-cholesterol, LDL-cholesterol, adiponectin, leptin, and TNF-α, as well as regulate lipid metabolism and reduce inflammation, was demonstrated in a systematic review studying the effects of probiotics in children with obesity [143].

Prebiotics are indigestible food elements that promote the growth of good bacteria in the host, hence improving host health. They are an intriguing treatment possibility for treating obesity. Probiotics not only promote the proliferation of beneficial bacteria but also boost the synthesis of SCFAs, which are crucial for the secretion of gut peptides [144]. Prebiotic supplementation can be regarded as a dietary intervention for preventing and treating T2D, and they may also aid in combating obesity by influencing food intake, appetite, and metabolic processes [145,146]. Prebiotics such as oligofructose appear to have satietogenic effects and regulate appetite in humans [147,148].

FMT has gained increasing attention in recent years, and studies have shown that it is effective in treating inflammatory bowel illnesses, irritable bowel syndrome, and certain neurological conditions [149]. More recently, researchers began to investigate the use of FMT for the treatment of obesity and its associated comorbidities because gut microbiota interventions can modulate glucose metabolism as well as SCFA production and reduce inflammation [150].

FMT ameliorates metabolic parameters in obese subjects, as demonstrated by several authors. In 2012, a small randomized controlled trial performed by Vrieze et al. demonstrated that FMT from lean donors to obese subjects led to a reduction in insulin resistance and an increase in SCFAs levels [29]. Similarly, Zecheng et al. showed in a meta-analysis and systematic review that FMT ameliorates blood glucose metabolism, insulin resistance, blood pressure, cholesterol levels, and SCFA levels and improves inflammatory responses in overweight patients [151]. It has been demonstrated that FMT raises the amount of *Akkermansia muciniphila*, which has been found to have positive impacts on obesity in both human and animal models [70,152]. Although FMT appears to have the capacity to ameliorate metabolic parameters, it has not been linked to weight loss in obese patients [44]. Moreover, the absence of standardized treatment plans, documented safety guidelines, and the possibility of patients attempting the process at home without medical supervision are major concerns surrounding FMT. Another concern is the lack of screening tests for donors particularly concerning the detection of pathogens or diseases, which leads to an increased risk of transmitting certain bacteria which may cause disease especially in immunocompromised subjects [153].

## 8. Conclusions and Perspectives

Recent studies have demonstrated that prolonged inflammation in the body, characteristic of obesity and diabetes, significantly impacts glucose metabolism, while maintaining eubiosis supports proper immune function. This suggests that implementing gut microbiota modulation strategies could offer a promising new approach to treating metabolic diseases. Probiotics are a relatively recent treatment option for obesity and other related disorders, but there are still many obstacles to overcome before probiotics can be widely used in clinical settings. Probiotics have a few restrictions when it comes to treating this illness. Research has indicated that the impact of probiotics on obesity varies according to the bacterial strain utilized, which makes it challenging to pinpoint a particular strain that is effective in the fight against obesity.

It is still difficult to figure out the best probiotic dosage and duration of administration in terms of treating obesity. The composition of each person’s microbiota, nutrition, and environmental circumstances are just a few of the variables that can affect how effective a probiotic is.

One of the most significant characteristics of the human gut bacterial community is the diversity of each person’s gut microbiota. Because of this, research has shown that, despite certain common aspects, it is exceedingly difficult to determine the precise way in which a given probiotic would interact with each person’s own microbial environment.

The field’s ongoing advancements regarding the interactions between probiotics and the gut microbiota not only shed light on established pathways but also pave the way for the discovery of novel ones.

It is challenging to present solid proof of the effectiveness of probiotics in treating obesity due to the wide range of study designs and quality issues.

To overcome these obstacles, a multidisciplinary strategy is needed, including ongoing research, standardized clinical trials to guarantee the validity and consistency of the results, and personalized medicine to customize probiotic treatments based on each patient’s unique gut microbiota profile and metabolic requirements.

If probiotic therapies can get past these obstacles, they might prove to be an effective complementary treatment for managing obesity.

Furthermore, FMT has shown promising results in ameliorating the anthropometric parameters in obese patients but has little effect on weight loss. Robust studies are, however, needed to enhance evidence-based knowledge and establish clear guidelines for the application of FMT in adults with overweight or obesity. Nevertheless, gut microbiota modulation techniques are not a substitute for a healthy lifestyle.

There are specific groups of population that are understudied, and more relevant information is needed to further enhance the knowledge on the topic and provide more depth to the current understanding of obese children, children from obese mothers, and obese mothers in general. The gut microbiota composition is comparable in obese adults and children under the age of ten in several ways. Compared to people of the same age who are not obese, both groups frequently display decreased microbial diversity, which is associated with a higher frequency of metabolic problems [154,155,156]. Additionally, several studies show that both adults and children have higher *Firmicutes*/*Bacteroidetes* ratios, supporting the notion that some dysregulated components of the gut microbiota contribute to the onset and maintenance of obesity [157,158]. Furthermore, some strains may be more common in the gut microbiomes of both groups, which may help with fat storage and energy extraction from the diet [159,160].

However, several distinctions should be noted between these two groups. Firstly, the gut microbiota of children is still developing at younger ages and is primarily influenced by factors such as nutrition, antibiotic exposure, and breastfeeding when applicable, whereas the microbiota of obese adults is generally stable and established [159,160]. Given this evidence, it can be concluded that children’s diets differ significantly from adults’, typically due to parental choices. This might result in microbial profiles that differ even in children who are obese [161,162].

Changes in the gut microbiota during pregnancy may have an impact on postpartum weight management as well as weight gain in the mother. The gut microbiota of pregnant women frequently alters, generally becoming less diverse [163,164]. Hormonal fluctuations, nutritional changes, and weight gain can all impact this shift. Pregnant women have a higher *Firmicutes*/*Bacteroidetes* ratio similar to obese adults. This could account for the rapid weight gain [165,166]. Pregnancy may increase the abundance of *Lactobacillus* and *Bifidobacterium* in the gut microbiota, which may have an impact on fat storage and food metabolism [167]. Due to their effects on appetite regulation, the dysbiosis’s tendency to increase inflammation, and metabolic problems during childbirth, these changes may make women more likely to retain weight after giving birth [168,169]. The fact that the mother’s microbiota can affect the baby’s gut microbiome during delivery and breastfeeding, thereby influencing the child’s risk of obesity later in life, is a crucial component of these pathological abnormalities in the pregnant woman’s gut [170]. Nevertheless, new studies are necessary to further expand the knowledge on this topic.

In summary, the gut microbiota plays a significant role in obesity and its related health issues through various mechanisms including metabolism, inflammation, and hormonal regulation, as shown in Table 2. Ongoing research is needed to better understand these interactions and develop targeted interventions for obesity management.

## Figures and Tables

**Table 1:** A systematic review synthesis of primary studies, sample size, methodologies, findings, and limitations.

Study Design	Year	Participant Type	Number of Subjects	Focus	Methods	Conclusions	Study Limitations	References
Cross-sectional study	2019	Humans	24 patients	To evaluate the role of the gut microbiota in the success rate of bariatric surgery	Fecal samples were analyzed using High Throughput Sequencing	The gut microbiota plays a role in the success rate of bariatric surgery through its interaction with the bile acids milieu	Limited number of patients. The optimal time for achieving the maximum benefits of the gastric bypass could not be determined	Gutiérrez-Repiso et al. [38]
In vivo	2019	Mice and humans	33 mice; 19 obese and 19 non-obese pediatric patients; and 26 obese and non-obese adult patients	To study the role of the miR-181 family in regulating adipose tissue function and metabolism	miRNA was analyzed in different tissues of mice fed a normal chow diet and obese mice fed a high-fat diet. BMI and blood samples were analyzed in pediatric patients. Adipose tissue samples were analyzed in adult patients	Gut microbiota-derived metabolites can regulate the miR-181 in white adipose tissue modulating the metabolism in response to dietary and environmental changes	Different environmental conditions. miRNA family have other independent effects on host metabolism, different from their effect on white adipose tissue	Virtue et al. [39]
Clinical trial	2019	Humans	1280 German adults: 633 lean non-diabetic, 494 obese non-diabetic, and 153 obese individuals with T2D from German population	To analyze the microbial taxonomic and functional profiles of the gut microbiota of lean non-diabetic, obese non-diabetic, and obese individuals with T2D	Patients were grouped according to medication, dietary intake, supplement usage, and nutrition, and were phenotypically characterized and analyzed using 16S rRNA amplicon and shotgun metagenomic sequencing	The study differentiated the microbial components of each metabolic disease and identified possible dietary and medication therapies	Limited power for a stratified analysis of specific taxa and microbial processes. Limited number of T2D patients	Thingholm et al. [23]
Observational study	2020	Humans	10 534 participants aged 20 to 99 years from the United States and the United Kingdom	According to earlier research, *Akkermansia* has a preventive impact against obesity and could be a viable probiotic. However, common characteristics like age, sex, and diet could confuse the above impact; this needs to be confirmed in a general population	Statistical analysis of the datasets from the American Gut Project	High relative abundance of *Akkermansia* is associated with low risk of obesity and the association declines with aging	There were no available clinical records for several variables, including cancer, hypertension, and metformin use. The results may not be as generalizable as they may be because they are based on people who followed a Western diet. Validation is advised in subjects following an Eastern diet, even when the diet type is changed (vegan or nonvegan). Furthermore, the classification of certain characteristics, such as smoking frequency, may introduce inaccuracies into the analysis because of the nature of self-reported data	Zhou et al. [40]
Pilot study	2020	Humans	16 women and 4 men with median pre-surgery BMI of 47.7 kg/m^2^	To study changes in the fecal microbiota and metabolites in patients undergoing gastric bypass	Fecal samples of obese patients obtained before and 1 week, 1 month, and/or 3 months after surgery were analyzed using shallow shotgun metagenomics and untargeted fecal metabolomics	Significant changes in the fecal microbiota and metabolites post-gastric bypass, with notable increase in alpha-diversity at 3 months and reduced caffeine metabolites, indoles, and butyrate	Small sample size and missing repeated samples	Yu et al. [41]
Longitudinal cohort study	2020	Humans	9 morbidly obese patients	To study the structure and function of the gut microbiota of obese patients before, 6 months, and 12 months after gastric bypass	Fecal sample analysis using 16S rRNA amplicon gene sequencing, gas chromatography mass spectrometry, liquid chromatography mass spectrometry, and nuclear magnetic resonance spectroscopy	The importance of spatial modifications in mucosal and fecal microbiomes after gastric bypass that correspond with persistent changes in fecal fermentation and bile acid metabolism, associated with improved metabolic outcomes	Small sample size	Ilhan et al. [42]
Longitudinal long-term study	2021	Humans	9	To study the variations in the fecal metagenome and metabolome of patients with severe obesity after bariatric surgery	Fecal and blood samples were collected before and four years after bariatric surgery and analyzed (biochemical, metagenomic, and metabolomic)	Bariatric surgery leads to variations in gut microbiota composition and fecal metabolome that last a long time and could be associated with the remission of obesity	Number of patients. Difficulties in the collection of stool samples. Unequal number of male and female	Juárez-Fernández et al. [34]
Randomized, double-blind, placebo-controlled clinical trial	2022	Humans	72 overweight humans	To study the association between the administration of *Lactobacillus curvatus* HY7601 and *Lactobacillus plantarum* KY1032 with reduced obesity through modulation of the human gut microbiome	2 groups: probiotic groups (used 1 × 1010 colony-forming units of HY7601 and KY10320) and a placebo group (used the same product without probiotics) for 12 weeks	*Lactobacillus curvatus* HY7601 and *Lactobacillus plantarum* KY1032 have anti-obesity effects by regulating the gut microbiota	No significant change in body fat percentage due to the limited cohort	Mo et al. [43]
Randomized clinical trial	2022	Humans	41 adult patients with obesity	To study if fecal microbiota transplantation from a lean donor has positive effects on body weight and the outcome of bariatric surgery	Fecal microbiota transplantation from a lean donor or autologous placebo was administered into the duodenum by gastroscopy before bariatric surgery was performed at 6 months after	Fecal microbiota transplantation did not affect pre- or postsurgical weight loss	Limited numbers of patients	Lahtinen et al. [44]
Randomized, double-blind, placebo-controlled clinical trial	2023	Humans	90 Saudi overweight or obese adults	To study the anti-obesity effect of probiotic administration	Biochemical markers measured through blood samples of 2 groups: placebo and probiotic (“MCP^®^ BCMC^®^ strains”) for 12 weeks	Multi-strain probiotics induced beneficial changes in the gut microbiota: reduction in weight and in systemic inflammatory state	Limited cohort size and population specificity. Adjustment of strain concentrations	Almaki et al. [45]

**Table 2:** The role of gut microbiota in obesity and its comorbidities through various mechanisms.

Mechanism	Observations About Mechanism Involvement in Obesity	Observations About Mechanism Involvement in Obesity-Related Comorbidities	References
Metabolism of nutrients	The gut microbiota is involved in the digestion and fermentation of indigestible carbohydrates, producing SCFAs, which regulate energy metabolism and fat storage		den Besten et al. [65]
Energy harvesting	Certain bacterial strains can enhance the efficiency of energy extraction from dietary sources, thus contributing to weight gain and obesity		Davis et al. [159]
Inflammation	Dysbiosis can lead to increased intestinal permeability and systemic inflammation	Inflammation is linked to obesity and its comorbidities such as T2D and insulin resistance	Scheithauer et al. [171]
Hormonal regulation	Gut microbiota can interfere with the secretion of appetite regulatory hormones, ghrelin and peptide YY, affecting hunger and satiety signals		Leeuwendaal et al. [172]
Fat distribution	Gut microbiota can influence where fat is stored in the body, increasing the risk of visceral fat accumulation	Visceral fat accumulation leads to the metabolic syndrome	Le Roy et al. [173]
Modulating gut microbiota through dietary changes, prebiotics and probiotics	Managing weight	Improving metabolic health	Dasriya et al. [174]

## Abbreviations

DNMTs = DNA methyltransferases; FIAF = fasting-induced adipose factor; FMT = fecal microbiota transplantation; FXR = farnesoid X receptor; GLP-1 = glucagon-like peptide-1; GPRs = G-protein-coupled receptors; LPSs = lipopolysaccharides; NAFLD = nonalcoholic fatty liver disease; ncRNA = non-coding RNA; PPARs = peroxisome proliferator-activated receptors; PYY = peptide YY; SCFAs = short-chain fatty acids; TGR5 = G-protein-coupled bile acid receptor 1; T2D = type 2 diabetes; TMAO = trimethylamine N-oxide.
